# MMS hotspots: a cross-sectional comparison of U.S. counties with and without Mohs micrographic surgery

**DOI:** 10.1007/s00403-023-02751-x

**Published:** 2023-12-07

**Authors:** Ajay Nair Sharma, Nicholas Peterman, Margit Juhasz, Melissa Shive

**Affiliations:** 1 Department of Dermatology, University of California, Irvine, 118 Med Surg I, Irvine, CA 92697 USA; 2 Carle College of Medicine, University of Illinois, Urbana-Champaign, USA

**Keywords:** Skin cancer, Epidemiology, Spatial analysis, Geospatial analysis, Nonmelanoma, Melanoma, Health disparities, Mohs, Mohs micrographic surgery, Medicare

## Abstract

Healthcare access greatly impacts skin cancer diagnosis and mortality rates. Recognition of current disparities in Mohs micrographic surgery (MMS) access can assist future policy and clinical decisions to correct them. For the years 2014–2018, the CPT codes for MMS (17,311 and 17,313) were counted on a per county level across the United States per the Medicare Centers for Medicare & Medicaid Services (CMS) Medicare Prescriber Database. Any county with 0 MMS CPT codes recorded were classified as “without MMS cases.” MMS “hotspots” were identified as counties that possessed a high average number of MMS cases compared to the national average, while also being surrounded by counties that possessed a low average number of MMS cases compared to the national average. Three thousand eighty-four counties in the United States were analyzed; 785 (25%) counties were designated as “with MMS cases” and 2301 (75%) “without MMS cases.” There were no significant differences in age, ethnicity distribution, or cost per enrollee between the two designations. 74% of counties with MMS cases were considered urban, while only 25% of those without cases were urban (*p* < 0.01). The median household income was markedly higher in counties with MMS cases ($71,428 vs. $58,913, *p* < 0.01). With respect to education, more individuals in counties with MMS cases possessed their General Education Development (GED) (89% vs. 86%, *p* < 0.01) or a college degree (30% vs. 19%, *p* < 0.01). Forty-nine counties were considered MMS “hotspots.” The density of MMS procedures varies greatly based on geography, maintaining the urban–rural disparity matched by the distribution of MMS surgeons. Additionally, there remains a wide income and educational gap between counties with and without MMS. Identifying MMS hotspots may facilitate further investigation into potential surgical access disparities.

## Introduction

Mohs micrographic surgery (MMS) is an essential service in the arsenal of treatments for non-melanoma skin cancers and increasingly for melanoma as well [1–5]. Prior epidemiologic studies have looked at the distribution of Mohs surgeons in the United States to identify trends in access and shown relative concentrations of dermatologic surgeons in coastal and urban areas [6, 7].

This study aims to better understand and characterize the demographic and geographic distribution of MMS services in the United States to identify opportunities to improve care delivery. Through the implementation of spatial autocorrelation, MMS “hot spots” were identified—counties that specifically had a high density of MMS cases surrounded by areas with few MMS cases, representing areas with uneven distributions of these surgical services and potential barriers to care.

## Materials and methods

### Medicare data

For the years 2014–2018, the Current Procedural Terminology (CPT) codes for the first stage of MMS (17,311 and 17,313) per 10,000 Medicare members were counted on a per county level across the United States. Data was obtained from the Medicare Centers for Medicare & Medicaid Services (CMS) Medicare Prescriber Database [8]. Any county with 0 MMS CPT codes recorded were classified as “without MMS cases.”

The demographics of each country were derived from the CMS Enrollment Dashboard [9]. The following variables were obtained for each county: average Medicare age, total Medicare population, race distributions, standardized Medicare cost per enrollee, median household income, number of those living under the federal poverty line, and number of those without a General Education Development (GED) or college degree. Counties were also stratified into “rural” or “urban” based on the 2013 United States Department of Agriculture Economic Research Service’s rural–urban continuum codes [10]. Comparison t-tests and Chi-squared analyses were performed to assess the significance of differences at the 0.05 level.

### Geospatial analysis

With the dataset parsed per county, GeoDa 1.20 (Chicago, USA) was utilized for geospatial cluster analysis and the identification of MMS “hotspots” [11]. Local spatial autocorrelation was applied to categorize counties into four groups defined by a set of two high/low modifiers: High–High, High–Low, Low–High, and Low–Low. In this case, the local indicator of spatial association (LISA) was Moran’s I, which fulfilled the two requirements of any LISA [12]:

- The LISA for each observation gives an indication of the extent of significant spatial clustering of similar values around that observation.
- The sum of LISAs for all observations serves as a global indicator of spatial association.

The first High/Low modifier was created when comparing the average number of MMS cases in a particular county to the average number of MMS cases nationwide per county. The second High/Low modifier indicated whether counties had a similarly high or low number of cases when compared to their neighbors, as determined by Moran’s I. Each county’s Moran’s I value served as a pseudo p-value to reject the null hypothesis that the county was spatially random. Thus, a positive value for I indicated that a county has neighboring counties with similarly high or low MMS cases, whereas a negative value for I indicated a county had neighbors with a dissimilar number of MMS cases. MMS hotspots were identified as counties that possessed a high average number of MMS cases compared to the national average, while also being surrounded by counties that possessed a low average number of MMS cases compared to the national average (High-Low).

Dane County, Wisconsin can serve as an example of an MMS hotspot. The national average of MMS cases per 10,000 Medicare members per county from 2014 to 2018 was 47.6, while Dane County possessed 220.8 MMS cases. Seven counties surround Dane County (Sauk, Green Lake, Dodge, Jefferson, Rock, Green, Iowa), and the average number of MMS cases in these surrounding counties was 0. Thus, Dane County was considered an MMS hotspot as it fulfilled both hotspot criteria: (1) it possessed more MMS cases than the national average (*p* < 0.05), (2) its surrounding eight counties possessed fewer MMS cases than the national average (*p* < 0.05). In this case, the University of Wisconsin-Madison Hospital is located in Dane County, the probable explanation as to why this county was identified as a hotspot. This process was conducted for every county in the United States.

## Results

**Fig. 1:**
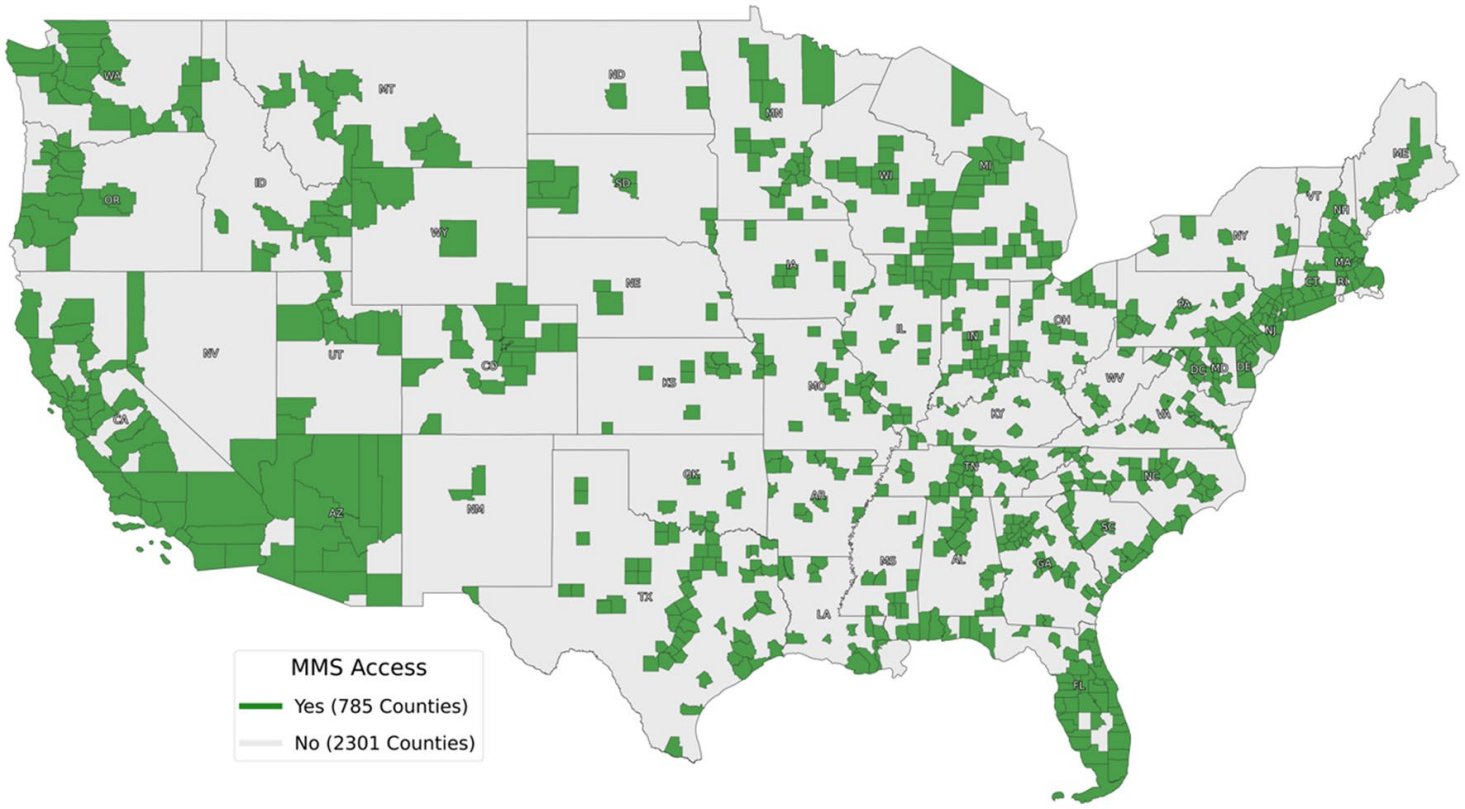
Geographic distribution of MMS medicare cases. Any county with “0” indicates that no MMS CPT codes were recorded, whereas a “1” indicates at least 1 MMS CPT code was recorded

A total of 3086 counties in the United States were analyzed; 785 (25%) counties were designated as “with MMS cases” and 2301 (75%) “without MMS cases” (Fig. 1). Geographically, 74% of counties with MMS cases were considered urban, while only 25% of counties without MMS cases were considered urban (*p* < 0.01) (major cities in the United States based on population having been overlayed on the national map for context). Similarly, counties with MMS cases were concentrated on both the West and East coasts, with 4 states possessing less than 5 counties with MMS cases (Nevada, New Mexico, North Dakota, Wyoming).

When comparing the demographics of each county, there were no significant differences in age or cost per enrollee between counties with and without MMS cases (Table 1). There existed a markedly higher Medicare population in counties with MMS cases (47,883 vs. 6325, *p* < 0.01), while the differences in racial distribution were extremely small, though statistically significant. Similarly, the median household income was higher in counties with MMS cases ($71,428 vs. $58,913, *p* < 0.01), with a lower proportion of individuals living under the federal poverty line (17.6 vs. 20.7%, *p* < 0.01). With respect to education, more individuals in counties with MMS cases possessed a GED or a college degree (89.1 vs. 86.2%, *p* < 0.01; 29.5 vs. 19.4%, *p* < 0.01, respectively).

**Table 1:** Demographic comparison of counties with and without MMS cases

Demographic variable	Counties with MMS Cases		Counties without MMS Cases		*p* value
	Mean	Standard deviation	Mean	Standard deviation	
Average medicare age (years)	71.23	1.43	71.26	1.99	0.67
Medicare population	47,883.6	80,355.9	6325.4	8178.0	< **0.01**
Male	45.48	1.82	46.56	2.44	< **0.01**
White	83.29	12.53	85.72	11.38	< **0.01**
Black	8.64	10.17	7.51	9.62	< **0.05**
Hispanic	4.16	6.91	3.53	6.98	< **0.05**
Other race	3.91	4.09	3.23	3.14	< **0.01**
Standardized medicare cost/enrollee ($)	9373.4	1259.2	9314.1	1486.7	0.32
Median household income ($)	71,428.1	19,248.5	58,913.6	13,191.6	< **0.01**
Urban (0,1)	0.74	0.44	0.25	0.43	< **0.01**
Poverty	17.6	6.51	20.69	7.94	< **0.01**
Without GED	10.92	4.82	13.82	6.51	< **0.01**
College degree	29.52	11.15	19.38	7.36	< **0.01**

*GED* general educational development, *MMS* Mohs micrographic surgery

**Fig. 2:**
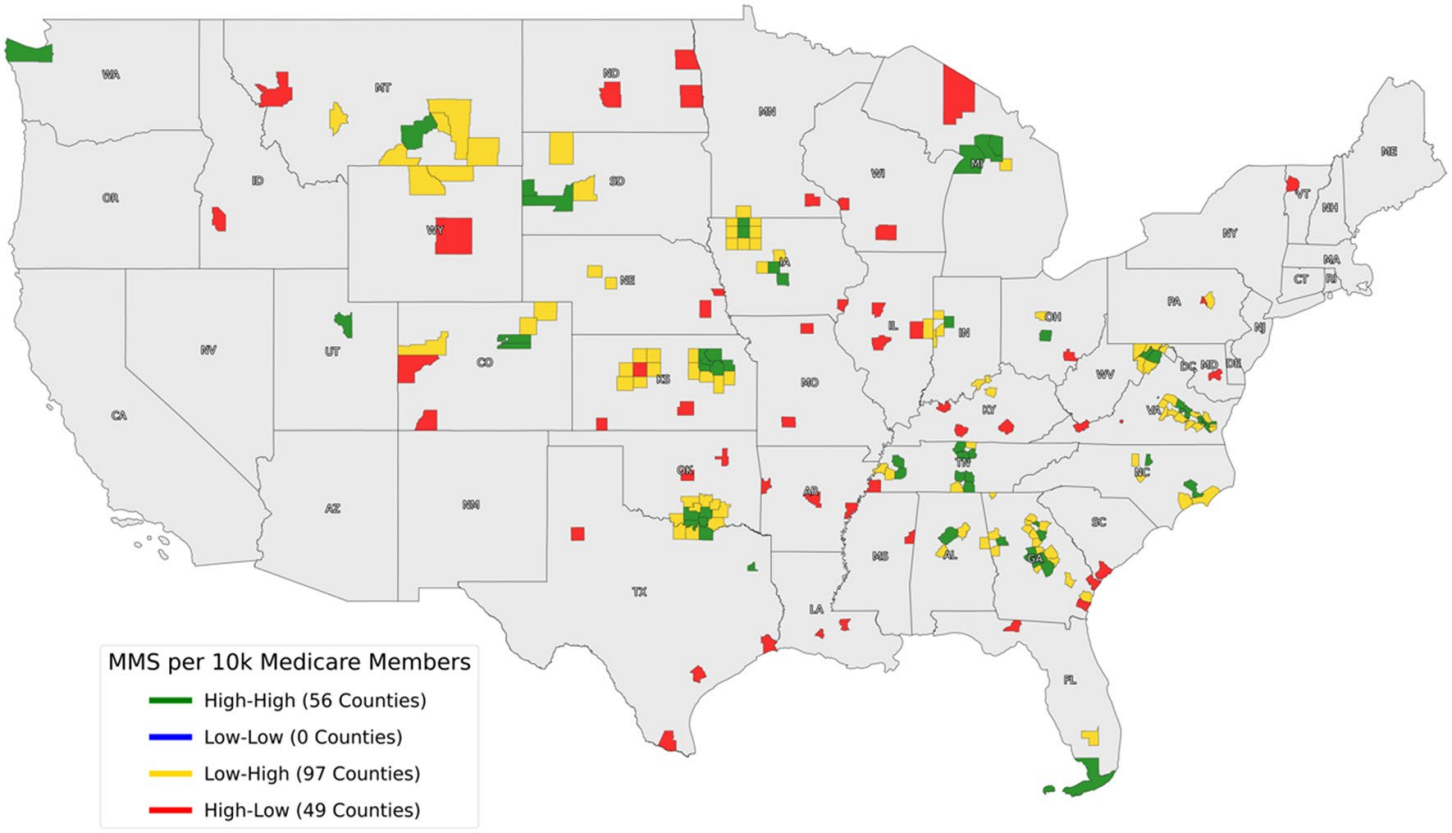
Cluster analysis of counties with MMS cases compared to the national average and average of their neighbors. MMS “hotspots” are indicated by “High-Low”

Forty-nine counties were identified as MMS hotspots per the outlined criteria (Fig. 2). There was a clear predilection for the central United States, with no hotspots identified on the entire West Coast.

## Discussion

Mapping the distribution of MMS cases throughout the nation, in addition to identifying counties with uniquely high relative volume, allows for a better understanding of MMS access. The vast majority of counties with MMS cases were considered urban, matching the large urban–rural disparity of dermatologists who perform MMS themselves [7]. Specifically, more fellowship-trained Mohs surgeons practice in urban areas, a potential result of more fellowship programs concentrated in populous areas with academic medical centers [13]. It is difficult to assess whether the dearth of MMS cases in most counties in some states indicates a shortage of Mohs surgeons in rural areas at present or an appropriate exodus of providers to higher-demand areas. However, there are data that support a metropolitan bias amongst practicing dermatologic surgeons, suggesting that rural areas face low surgical access rather than low demand, just as is seen with general dermatology, resulting in longer travel distances, wait times, and reduced access [7]. With the demand for medical dermatologists and Mohs surgeons increasing due to an aging population and rising rates of skin cancer, initiatives may be required to incentivize dermatologists to practice in underserved areas [14].

Demographic differences between counties with and without MMS cases exist as well, though their significance is unclear. Counties with MMS cases possess more formally educated citizens with respect to both GED (3% higher) and college degrees (10% higher). Additionally, the average household income in counties with MMS cases was $12,515 higher than that in counties without MMS cases. Both of these gaps in educational attainment and income may have a relationship with differences in sun exposure, skin cancer risk factors, or access to dermatologic care, subsequently manifesting as inequalities in MMS cases [15].

Through the identification of MMS “hotspots”, it is possible to hypothesize factors that may affect surgical location preference. Possible explanations include the presence of academic medical centers (e.g. Natrona County, Wyoming) or fellowship-trained Mohs surgeons (*e.g.* Lubbock, Texas) in largely rural areas [7]. MMS cases may need to be concentrated in only these few counties throughout the state due to a smaller total size of the patient population, need for complex surgical repair possible only at an academic center, or provider personal and financial reasons. Conversely, it is imaginable that states with other forms of healthcare systems and no MMS hotspots (e.g. managed care organizations in Arizona) possess a more equal distribution of MMS cases given the creation of more numerous Mohs surgery sites. Using these states as an example, novel forms of healthcare delivery may allow for increased access across counties with a lower number of fellowship-trained Mohs surgeons.

It is also important to acknowledge the differences between revealing MMS hotspots and simply mapping the distribution of Mohs surgeons in the United States, as hotspots reveal locations where patients are particularly reliant on surgical care. Since patients in neighboring counties have a lower number of cases relative to the hotspot county, hotspots may serve as one of the few options for MMS, necessitating patients to make logistic accommodations (i.e. facilitating transportation, ensuring insurance coverage) to receive treatment. Additionally, not every isolated county with MMS cases was considered a hotspot (ex: Orange County, California) given their average number of cases was not higher than the national average, indicating the possible presence of particularly attractive factors in hotspot counties.

There are several limitations to this study worth considering. First and foremost, the data in this study is primarily descriptive and it is not possible to draw causal conclusions as to why these hotspots exist. While it is plausible hotspots exist due to inequities in care as suggested, it may also be the case that they represent the most practical and efficient method of providing access to MMS to people living in sparsely populated rural counties. Second, this is a retrospective dataset that is reliant on correct CPT coding, and it is possible that many MMS cases within the time frame of this study were either billed incorrectly or not billed at all. It is also important to remember that this dataset is based on billing data and is not a substitute for tumor incidence and also does not reflect whether tumors were appropriately or inappropriately treated with MMS or should have been treated with MMS but were not [16]. Lastly, given this study includes data only from the Medicare population, conclusions made towards the general population must be applied with caution. Additional study is needed to further analyze MMS trends and the existence of MMS hotspots among the nation’s entire population.

## Conclusion

From a comprehensive analysis of 2014–2018 Medicare data, it is clear that the density of MMS procedures varies greatly based on geography, maintaining the urban–rural disparity matched by the distribution of Mohs surgeons [7, 17]. There also remains a wide income and educational gap between counties with and without MMS. By searching for the presence or absence of MMS hotspots and further investigating the reasons for them, providers may find insight into why specific counties provide an unexpectedly high number of surgical cases.

## Data Availability

The data that support the findings of this study are available from the first author, ANS, upon reasonable request.
